# Hereditary nonspherocytic hemolytic anemia caused by glucose-6-phosphate isomerase (GPI) deficiency in a Chinese patient: a case report

**DOI:** 10.1186/s12887-022-03522-9

**Published:** 2022-08-01

**Authors:** Yumei Zu, Hui Wang, Weijia Lin, Chaochun Zou

**Affiliations:** 1 Department of Endocrinology, the Children’s Hospital of Zhejiang University School of Medicine, No. 3333 Binsheng Road, Hangzhou, 310057 China; 2 Department of Rehabilitation, the Children’s Hospital of Zhejiang University School of Medicine, Hangzhou, 310057 China

**Keywords:** Glucose-6-phosphate isomerase (GPI) deficiency, Hereditary nonspherocytic hemolytic anemia (HNSHA), Jaundice

## Abstract

**Background:**

Glucose phosphate isomerase (GPI) deficiency is a rare autosomal recessive disorder that causes hereditary nonspherocytic hemolytic anemia (HNSHA). Homozygous or compound heterozygous mutation of the GPI gene on chromosome 19q13 is the cause of GPI deficiency. Fifty-seven GPI mutations have been reported at the molecular level.

**Case presentation:**

A 5-month-old boy was presented with repeated episodes of jaundice after birth. He suffered from moderate hemolytic anemia (hemoglobin levels ranging from 62 to 91 g/L) associated with macrocytosis, reticulocytosis, neutropenia, and hyperbilirubinemia. Whole-exome sequencing showed that he has a missense mutation c.301G > A (p.Val101Met) in exon 4 and a frameshift mutation c.812delG (p.Gly271Glufs*131) in exon 10. Mutation p.Gly271Glufs*131 is a novel frameshift null mutation in GPI deficiency.

**Conclusion:**

In a patient with recurrent jaundice since birth, mutations in the GPI gene associated with HNSHA should be evaluated. The c.812delG (p.Gly271Glufs*131) variant may be a novel mutation of the GPI gene. Compound heterozygous mutations c.301G > A (p.Val101Met) and c.812delG (p.Gly271Glufs*131) are not relevant to neurological impairment.

## Background

Glucose-6-phosphate isomerase (GPI) deficiency (MIM 613470), one of hereditary nonspherocytic hemolytic anemias (HNSHA), is a rare autosomal recessive hereditary disease caused by homozygous or compound heterozygous mutations of GPI gene on chromosome 19q13 [1]. Although GPI deficiency is the second most common erythro-enzymopathy of anaerobic glycolysis after pyruvate kinase deficiency, its exact morbidity is not known yet [2]. About 90 patients have been reported, to date, from a variety of ethnic groups and populations throughout the world since the first report in 1968 by Baughan et al. [3]. GPI deficiency is characterized by mild-to-severe chronic hemolytic anemia, jaundice, splenomegaly, and an increased incidence of pigment gallstones and cholecystitis, which is mainly caused due to dysregulated catalyzation of the second step of glycolysis [4]. According to a few reports, the fate of such patients was fetal loss/hydrops fetalis or immediate neonatal death [5–10]. In addition to its essential role in carbohydrate metabolism, GPI is identical to neuroleukin, a neurotrophic factor that supports the survival of embryonic spinal neurons, skeletal neurons, and sensory neurons [4, 11]. Therefore, some patients also showed muscle weakness, mixed sensory and cerebellar ataxia, mental retardation, or epilepsy [6, 7, 9, 12–23]. GPI also functions as a tumor-secreted cytokine and an angiogenic factor, which helps in the stimulation of endothelial cell motility [24]. Diagnosis is based on the determination of the enzymatic activity of GPI in erythrocytes using a quantitative assay and confirmation by DNA sequence analysis of the GPI gene [11, 24]. Treatments for chronic hemolytic anemia include blood transfusions, splenectomy, and supportive therapy. Here, we reported a patient with compound heterozygous mutation of the GPI gene who presented chronic hemolytic anemic features and reviewed correlative literature.

## Case presentation

A 5-month-old Chinese boy presented to our unit because of repeated episodes of jaundice after birth. He was noted to be markedly anemic at the age of 3 hours at the local hospital. For the next 5 months, he suffered from moderate hemolytic anemia (Hemoglobin 62–91 g/L), along with macrocytosis (mean corpuscular volume 95–116 fL), reticulocytosis (461–489 × 10^9^ /L reticulocytes), and neutropenia (neutrophils 0.53–2.25 × 10^9^ /L) (Table 1), and unconjugated hyperbilirubinemia (133 μmol/L). He also suffered from active bone marrow hyperplasia with erythroid hyperplasia and neutropenia (granulocytes = 17.6%, erythrocytes = 41.2%, granulocytes: erythrocytes = 0.43:1) as assessed by analyzing the blood smear and bone marrow cytomorphology. Hemolytic anemia was considered, and blood transfusion (once, at 56-day-old) and other treatments, including protein iron succinate oral solution, vitamin C, vitamin B12, and prednisone acetate, were administered at the local hospital. He was born at 38 weeks with a birth weight of 3.07 kg. His parents were not consanguineous and had no history of anemia before.

**Table 1:** Blood parameters of the patient

Parameters \ Age	1 m, 23 d	1 m, 26 d	3 m	4 m	5 m
Red blood cells (× 10^12^ /L)	1.90	2.87	2.34	2.15	2.55
White blood cells (× 10^9^ /L)	3.60	3.19	7.44	5.27	10.86
Neutrophils (× 10^9^ /L)	0.53	0.57	0.61	0.54	2.25
Platelets (× 10^9^ /L)	267	289	405	464	646
Hemoglobin (g/L)	62	90	72	73	91

On physical examination, he had a mild anemic appearance and a bodyweight of 6.1 kg. The liver was palpated about 2 cm below the right costal margin while the spleen was not palpated below the left costal margin. The chest, cardiac, abdominal, nerve, and skin were unremarkable. Cardiac ultrasonography indicated patent foramen ovale. Other tests, such as erythrocyte osmotic fragility test, hemoglobin electrophoresis, direct Coombs test, blood gas, electrolyte, chest X-ray, electrocardiogram, and ultrasonography for the brain, abdominal, and pelvic organs, showed normal results.

**Fig. 1:**
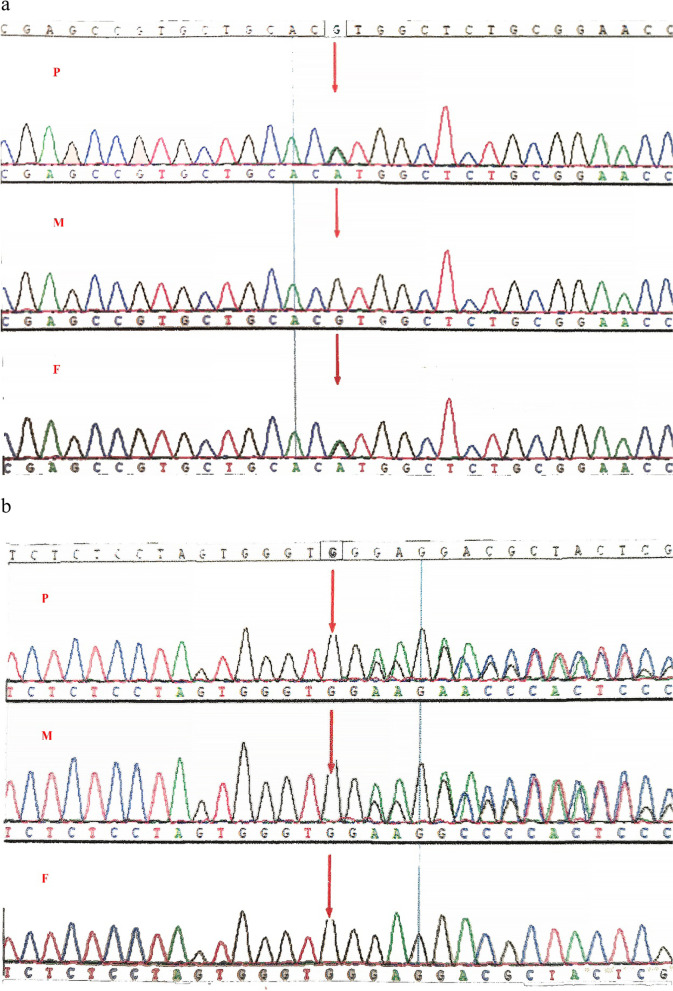
Sanger sequencing results of our patient and his parents. The red arrow indicates the variant locus. **a** A c.301G > A (p.Val101Met) hemizygous mutation in the GPI gene originated from the patient’s father. **b** A c.812delG (p.Gly271Glufs*131) hemizygous mutation in the GPI gene originated from the patient’s mother. P, proband; M, mother; F, father

For etiology, whole-exome sequencing was performed. The outcome demonstrated that the patient had a c.301G > A (p.Val101Met) mutation in exon 4 that originated from his father and a c.812delG (p.Gly271Glufs*131) mutation in exon 10 that originated from his mother, both of which were specific compound heterozygous variants of the GPI gene. No other variants associated with hereditary hemolytic anemia were detected. The GPI variants were identified using Sanger sequencing (Fig. 1). The missense mutation c.301G > A (p.Val101Met) was previously reported in an Italian male patient (GPI Sarsina) who is homozygous and a female patient who is compound heterozygous with c.1009G > A (p.Ala337Thr) [2, 25, 26]. However, the frameshift is not reported in the ClinVar database, the Human Gene Mutation Database, and the Leiden Open Variation Database. The two variants observed in our patient were regarded as pathogenic according to the criteria by the American College of Medical Genetics and Genomics.

## Discussion and conclusions

Our patient was characterized by intermittent jaundice along with unconjugated hyperbilirubinemia and moderate hemolytic anemia associated with macrocytosis, reticulocytosis, and neutropenia, which were characteristics of chronic HNSHA. He did not have typical splenomegaly, which might be an advantage of his previous therapy or may appear later on. Uncertain chronic hemolytic anemia is supposed to be excluded from other erythropathies so that patients can receive treatments timely, and that reveals more potential changes. Previous reports have recommended pertinent workflows for the diagnosis of chronic hemolytic or inherited anemias [18]. Given the normal shape of erythrocytes, normal hemoglobin, and the negative results of the direct Coombs test, we assumed that our patient has an erythro-enzymopathy instead of sickle cell disease, thalassemia, or autoimmune conditions. We regret that enzymatic assays were not performed; however, the diagnosis of GPI deficiency using a biochemical method is unclear [24]. Bioinformatic analysis implied that both GPI variants were deleterious mutations. Thus, we concluded that the patient suffered from the GPI deficiency. We suggested a splenectomy and other symptomatic treatments, like fluid infusion, blood transfusions, vitamin supplementation when needed, and prevention of infections due to exogenous oxidation agents and the use of drugs, which were found to be suitable for other GPI deficiency patients [2, 27]. It is noted that transfusion-dependent patients can benefit from splenectomy [2, 4].

The GPI gene is located on chromosome 19q13.1, contains 18 exons, and its cDNA of 1.9 kb codes for 558 amino acids [4]. To date, 57 GPI mutations have been reported at the molecular level (The Human Gene Mutation Database, http://www.hgmd.cf.ac.uk, accessed on 12 Feb 2022), which includes 53 missense/nonsense, one splicing, and 3 small deletions [28]. Although our patient is the 8th case of GPI deficiency in China since the first report in 1992 by Zhao et al. [29], our patient is only the 4th case where genetic confirmation was performed (Table 2). Our patient has a missense mutation c.301G > A (p.Val101Met) and a frameshift mutation c.812delG (p.Gly271Glufs*131). The fact that the mutation p.Val101Met was identified in two different ethnic groups implied that either the origin of the mutation is very old or that the same mutation arose in more than one individual. A study of the GPI polymorphisms may be helpful in elucidating whether this mutation has a single origin. A targeted next-generation sequencing clinical panel of GPI genes can expedite molecular diagnosis rather than using Sanger sequencing in such cases [24]. The mutation p.Val101Met was first found in the Chinese population. Further knowledge regarding the GPI polymorphisms may help to draw further conclusions.

**Table 2:** Mutations reported in the Chinese patients with GPI deficiency

Case	Exon	Mutation	Consequence	Zygosity	Reference
1	7 18	c.637 T > A c.1614C > G	p.Phe213Ile p.His538Gln	Compound heterozygous	[19]
2	6	c.553 T > A	p.Phe185Ile	Homozygous	[21]
3	6 10	c.490C > A c.817C > T	p.Pro164Thr p.Arg273Cys	Compound heterozygous	[22]
4	4 10	c.301G > A c.812delG	p.Val101Met p.Gly271Glufs*131	Compound heterozygous	current case

GPI plays an important role in physiological activities in addition to its essential role in the energy pathway, and it is present in all living organisms and expressed in all tissues [20]. The enzyme is homodimeric, traditionally termed large and small domains [30]. Several missense mutations in the GPI gene induce protein abnormalities that influence the enzyme catalytic activity [30]. Previous studies have analyzed the amino acid substitution p.Val101Met. It is located in the α7 helix of its big domain, where the longer side chain of methionine might decrease the local packing efficiency and a protein destabilization through a clash with the phenyl group of F85 between α6 and α7 [31]. Alternatively, it also potentially disrupts the active site architecture by altering the critical interactions between α6, α7, and α15 and the 3/10 helix between residues 270 and 274, which is one of the active sites [30]. The neurological symptoms were absent in the three patients, suggesting that the neurotrophic activity of the GPI enzyme is not affected by the amino acid substitution p.Val101Met. Compared to patient GPI Sarsina, our patient has the frameshift mutation c.812delG (p.Gly271Glufs*131), which disrupts the open reading frame of the GPI mRNA transcript, predicting the formation of a truncated polypeptide, altering active site architecture (residues 270–274), and lacking about 28% of the COOH terminal amino acid sequence and the active site 519. As a result, this abnormal polypeptide may not be compatible with dimerization, suggesting that our patient can be considered functionally hemizygous for the missense mutation (p.Val101Met) present in the other chromosome. In other words, this frameshift mutation can be assessed as potentially pathogenic.

The current report describes the clinical features and the molecular etiology of a Chinese patient with GPI deficiency, a very rare cause of HNSHA. The patient was compound heterozygous for the novel GPI frameshift mutation p.Gly271Glufs*131 and missense mutation p.Val101Met. Molecular structural analysis suggested that both variants affect the active site of the enzyme but do not interfere with its neurotrophic properties.

## Data Availability

The variant data that support the findings of this study have been deposited in ClinVar with the SCV accession codes, SCV002549741 and SCV002549742.

## Abbreviations

GPI: Glucose-6-phosphate isomerase
HNSHA: Hereditary nonspherocytic hemolytic anemia
